# Clinical outcomes associated with evolving treatment modalities and radiation techniques for base-of-tongue carcinoma: thirty years of institutional experience

**DOI:** 10.1002/cam4.364

**Published:** 2015-01-26

**Authors:** Leechuan Andy Chen, Christopher J Anker, Jason P Hunt, Luke O Buchmann, Kenneth F Grossmann, Kenneth Boucher, Li-Ming Christine Fang, Dennis C Shrieve, Ying J Hitchcock

**Affiliations:** 1 Department of Radiation Oncology, Huntsman Cancer Hospital, University of UtahSalt Lake City, Utah; 2 Division of Otolaryngology, Head and Neck Surgery, Department of Surgery, University of UtahSalt Lake City, Utah; 3 Division of Oncology, Department of Internal Medicine, University of UtahSalt Lake City, Utah; 4 Study Design and Biostatistics Center, Division of Epidemiology and Public Health, Department of Internal Medicine, University of UtahSalt Lake City, Utah; 5 Department of Radiation Oncology, University of WashingtonSeattle, Washington

**Keywords:** Base-of-tongue carcinoma, brachytherapy, chemotherapy, intensity-modulated radiotherapy (IMRT), surgery

## Abstract

Curative treatment for base-of-tongue squamous cell carcinoma (BOT SCC) has evolved over time; however, comparative outcomes analysis for various treatment strategies is lacking. The authors reviewed the evolution of treatment modality and radiotherapy (RT) technique for 231 consecutive BOT SCC patients at our institution between 1981 and 2011. Treatment modalities included definitive chemoradiotherapy (chemoRT) (42%), definitive RT (33%), surgery followed by RT (20%), and surgery alone (5%). RT techniques included external beam plus interstitial brachytherapy (EBRT + IB) (37%), conventional EBRT (29%), intensity-modulated radiation therapy ± simultaneous integrated boost (IMRT ± SIB) (34%). Clinical characteristics and outcomes were stratified by modality or RT technique. Treatment modality evolved from definitive RT (1980s–1990s) to definitive chemoRT (1990s–2000s). RT technique evolved from EBRT + IB (1980s–1990s) to conventional EBRT (1990s–2000s) to IMRT + SIB (2000s). With median alive follow-up of 6 years (0.3–28 years), the 5-year LC, LRC, and OS rates were 80%, 73%, and 51%. There was no difference in distribution of gender, age, stage among treatment modalities. Definitive chemoRT had improved LRC (HR 1.6) and OS (HR 1.7) compared to definitive RT. IMRT + SIB had improved LRC (HR 3.2), DFS (HR 3.4), and OS (HR 3.0) compared to conventional EBRT. Over the past 30 years, BOT SCC treatment has undergone major paradigm shifts that incorporate nonsurgical functional preservation, concurrent chemotherapy, and advanced RT techniques. Excellent locoregional control and survival outcomes are associated with accelerated IMRT with chemotherapy.

## Introduction

The curative treatment modality for locally advanced base-of-tongue squamous cell carcinomas (BOT SCC) has evolved over time. Historically, BOT SCC was treated with surgery followed by adjuvant RT if indicated. However, radical surgery is associated with swallowing and speech dysfunction as well as a high risk of local recurrence due to the limitation of wide local excision 1,2. Surgery with or without postoperative RT may be appropriate for small primary disease, but it is associated with poor outcomes for deeply infiltrated tumors 1–4.

To obtain optimal functional preservation, RT alone has become increasingly used as a definitive modality for oropharyneal carcinomas 5,6. Although primary RT has achieved functional preservation, conventional EBRT alone has yielded less satisfactory local control (LC) rates in locally advanced disease 7. To improve LC rates, different techniques have evolved to intensify the RT dose at the BOT tumor site. These include dose escalation using interstitial brachytherapy (IB) with conformal EBRT, accelerated or altered fractionation, and radiosensitization with the addition of chemotherapy. IB had been used to boost high RT doses to the tumor while sparing normal tissues, and had been used for decades in combination with EBRT for the treatment of advanced BOT SCC, with reported locoregional control (LRC) rates of 70–90% 8–10. Accelerated or altered fractionation has been shown to improve LRC 11,12. Addition of concurrent chemotherapy has been shown to improve LRC, although the survival data are still to be confirmed 13,14. More recently, the development of intensity-modulated radiation therapy (IMRT) has led to the integration of accelerated fractionation, dose escalation, and concurrent chemotherapy in the treatment of BOT SCC 15–17. There is paucity of data comparing the clinical outcomes of these various techniques. At our institution, the treatment of BOT SCC has incorporated evolving modalities and RT techniques over 30 years, reflecting a historic change in practice patterns. The purpose of this study was to review the evolution of treatment modalities and RT techniques for BOT SCC, and to compare the clinical outcomes associated with various treatment modalities and RT techniques.

## Methods and Materials

### Patient population

The institutional review board at the University of Utah approved the study. Patients with BOT SCC (ICD9 141.0) were identified using Tumor Registry and department database. The study entry criteria included adults greater than 18 years old with a biopsy-proven SCC of the BOT who were consecutively treated at our hospital with curative intent between February 1981 and December 2011. The exclusion criteria included incomplete RT, history of previous head and neck RT, death while on treatment, or distant metastases (stage IVC) at diagnosis. Nonsquamous cell carcinomas or primary tumors centered at other sites (oral cavity, hypopharynx, or larynx) that extended to the base of tongue were also excluded. All tumors were restaged according to the American Joint Committee on Cancer 2009 staging system. Patient, tumor, treatment characteristics, and follow-up data were collected for all patients.

### Treatment outlines

All patients received treatment at the Department of Radiation Oncology and/or Division of Otolaryngology-Head and Neck Surgery at the University of Utah. Patients were stratified by treatment decade, treatment modality, and RT technique. The three treatment decades were 1981–1990 (1980s), 1991–2000 (1990s), and 2001–2011 (2000s). Primary treatment modalities included surgery alone (local resection or composite resection), surgery followed by postoperative RT (with or without chemotherapy), definitive RT, and definitive chemoRT. RT techniques included EBRT followed by IB boost (EBRT + IB), EBRT alone by 2D or 3D conformal RT, accelerated RT or accelerated concomitant boost RT, and accelerated IMRT with simultaneous integrated boost (IMRT + SIB).

### Surgery plus postoperative RT

Surgical techniques included transoral CO_2_ laser or local excision for early-stage disease or composite resection for advanced disease. Postoperative RT was delivered by 2D, 3D, or IMRT techniques.

### EBRT plus interstitial brachytherapy

Initial comprehensive EBRT was delivered to the head and neck, followed by IB boost to the BOT primary tumor at 2–4 weeks after the completion of EBRT. The median total dose of EBRT + IB to the primary tumor was 75 Gy (range, 50–89 Gy), with a median EBRT dose of 50 Gy (range, 30–66 Gy) and a median IB dose of 25 Gy (range, 20–35 Gy). The technique of IB was previously described 9 and planned neck dissection was performed at the time of catheter implant. IB was delivered via either low dose rate (LDR) or high dose rate (HDR). LDR was delivered in one treatment over 2–3 days. HDR was delivered using an afterloading device, with the prescribed dose delivered using 5–6 Gy per fractions twice daily with at least 6 hours between fractions to a total dose of 30 Gy.

### Definitive EBRT alone

Definitive EBRT was delivered using conventional 2D or 3D conformal RT techniques, as described previously 18. Median EBRT dose to the BOT tumor was 70 Gy at 2 Gy per fraction given within 7 weeks (standard fractionation).

### Accelerated RT, accelerated concomitant boost RT, and IMRT + SIB

Accelerated RT was given 70 Gy at 2 Gy per fraction, six treatments a week, and completed within 6 weeks. Accelerated concomitant boost RT was delivered as a total dose of 72 Gy, 1.8 Gy per fraction for 30 treatments, and 1.5 Gy to boost the gross disease starting on fraction 19. The details of IMRT + SIB technique at our institution were previously published 17. Dose prescriptions for accelerated IMRT + SIB were 67.5 Gy at 2.25 Gy per fraction to gross tumor volume (GTV) plus margins, 60 Gy at 2 Gy per fraction to high-risk nodal sites, and 54 Gy at 1.8 Gy per fraction to elective nodal sites in 30 fractions.

### Outcomes analysis

Patients with <3 months of follow-up were censored in the analysis of treatment outcomes. Treatment failures were defined as persistent local disease, persistent nodal disease, local recurrence, regional nodal recurrence, or distant recurrence. Date of failure was the date of tissue confirmation or, if no tissue confirmation, the date of clinical/imaging exam showing clear evidence of failure. Date of death was determined from the Social Security Death Index and from medical records. Disease control and survival times were calculated from completion of primary treatment to date of failure or date of death or last follow-up. Late toxicity was defined as adverse events occurring at least 6 months after the completion of treatment. Severity was scored using CTCAE v4.0 and ≥grade 3 toxicities were recorded.

### Statistical analysis

Statistical analysis was performed using StatsDirect statistical software (version 2.78; Stats Direct Ltd., Altrincham, UK). Differences in characteristics and toxicity were detected using the Fisher's exact test. LC, regional control (RC), LRC, distant metastases-free survival (DMFS), disease-free survival (DFS), and overall survival (OS) rates were estimated using the Kaplan–Meier method. Disease control and survival were calculated from completion of treatment. Log-rank analysis was used for the comparison of rates among groups. Statistical significance was set at *P* < 0.05.

## Results

### Clinical characteristics

Table1 describes characteristics of this cohort. Two hundred and nine (93%) patients had stage III or IV disease, with 118 patients (51%) having T3–T4 disease and 149 patients (65%) having N2b or higher nodal metastases. Stratified by treatment modality, 97 patients (42%) received definitive chemoRT, 76 (33%) had definitive RT, 46 (20%) had surgery followed by postoperative RT, and 12 (5%) had surgery alone. Among those who received definitive RT, 64 (37%) had EBRT + IB, 50 (29%) had 2D or 3D EBRT, 45 (26%) had IMRT + SIB, and 14 (8%) had IMRT without SIB.

**Table 1:** Patient, disease, and treatment characteristics

Characteristics	*n*	% total
Gender		
Male	204	88%
Female	27	12%
Age		
Median (range)	59	(32–88)
T stage		
T1	38	16%
T2	75	32%
T3	33	14%
T4a	79	34%
T4b	6	3%
N stage		
N0	35	15%
N1	36	16%
N2a	11	5%
N2b	75	32%
N2c	50	22%
N3	24	10%
Stage group		
I	9	4%
II	7	3%
III	33	14%
IVA	156	68%
IVB	26	11%
Treatment modality		
Definitive chemoRT	97	42%
Definitive RT	76	33%
Surgery + postop RT	46	20%
Surgery only	12	5%
Primary RT techniques		
EBRT + IB	64	37%
EBRT (2D/3D)	50	29%
IMRT + SIB	45	26%
IMRT	14	8%

EBRT±IB, external beam plus interstitial brachytherapy; RT, radiotherapy; IMRT ± SIB, intensity-modulated radiotherapy with simultaneous integrated boost.

Table2 describes characteristics stratified by the treatment modalities for stage III or IV patients. There were no significant differences in distribution of gender, age, T-stage, N-stage, or overall stage among the three predominant modalities. Table3 describes characteristics stratified by definitive RT techniques for stage III or IV patients. Factors that differed by RT techniques were T-stage and concurrent chemotherapy. Patients who received EBRT + IB had a higher proportion of T3/T4 disease, compared with those who received conventional EBRT or IMRT + SIB. Patients who received IMRT + SIB more frequently received concurrent chemotherapy, compared with those who received EBRT + IB or conventional EBRT.

**Table 2:** Characteristics stratified by treatment modality for stage III–IV patients

Characteristics	Definitive chemoRT (*n* = 96)	Definitive RT *n* = 69)	Surgery + RT (*n* = 45)	*P* value
Gender				
Male	92	60	38	NS
Female	4	9	7	
Age				
≤60 years old	56	34	29	NS
>60 years old	40	35	16	
T stage				
T1/T2	41	26	27	NS
T3/T4	55	43	18	
N stage				
N0–N1	19	20	14	NS
N2–N3	77	49	31	
Stage group				
III	11	10	10	NS
IVA	74	47	32	
IVB	11	12	3	
Concurrent chemotherapy				
Yes	96	0	17	n/a
No	0	69	28	

RT, radiotherapy.

**Table 3:** Characteristics stratified by RT techniques for stage III–IV patients

Characteristics	EBRT + IB (*n* = 57)	EBRT (2D/3D) (*n* = 49)	IMRT + SIB (*n* = 45)	*P*-value
Gender				
Male	48	44	45	NS
Female	9	5	0	
Age				
≤60 years old	31	24	25	NS
>60 years old	26	25	20	
T stage				
T1/T2	15	20	25	0.01
T3/T4	42	29	20	
N stage				
N0–N1	20	9	8	NS
N2–N3	37	40	37	
Stage group				
III	12	4	4	NS
IVA	34	37	38	
IVB	11	8	3	
Concurrent chemotherapy				
Yes	15	23	44	<0.01
No	42	26	1	

IMRT ± SIB, intensity-modulated radiotherapy with simultaneous integrated boost; RT, radiotherapy; EBRT±IB, external beam plus interstitial brachytherapy.

### Evolution of treatment modalities and RT techniques

**Figure 1:**
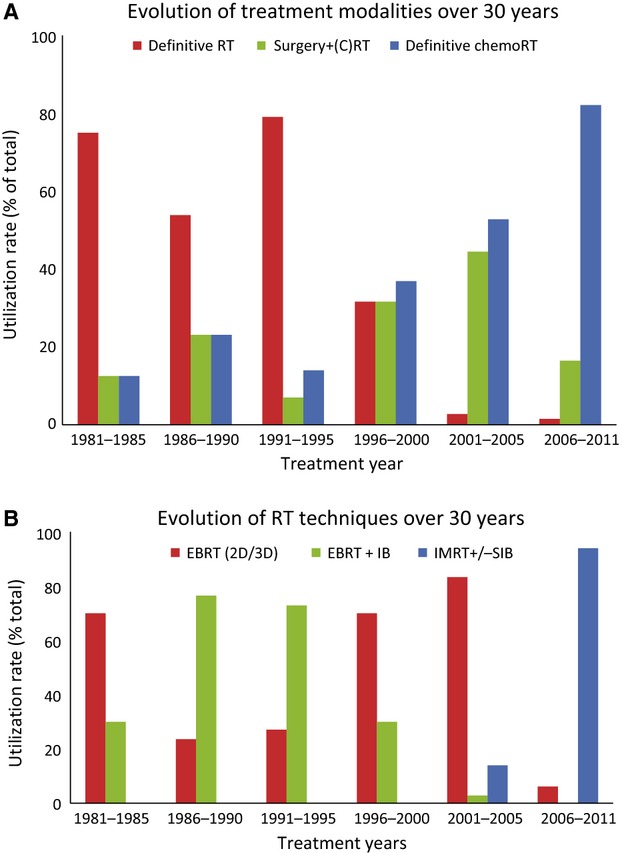
(A) Evolution of treatment modalities over 30 years. (B) Evolution of RT techniques over 30 years. RT, radiotherapy.

The treatment period spanned three decades, with 43 patients treated in the 1980s, 91 patients treated in the 1990s, and 97 patients treated in the 2000s. Figure1A shows utilization rates of different treatment modalities over 30 years. RT alone was the predominant treatment modality between 1981 and 1995. Its utilization rate drastically declined over the next 10 years from 79% (1991–1995) to 32% (1996–2000) to 3% (2001–2005). Conversely, concurrent chemoRT increased from 14% (1991–1995) to 37% (1996–2000) to 53% (2001–2005) to 82% (2006–2011). The most common chemotherapy regimen in the 1980s was cisplatin (100 mg/m^2^) and 5-FU (1000 mg/m^2^ continuous infusion for 96 h) given every 3 weeks for two to three cycles. Cisplatin single agent at 100 mg/m^2^ every 3 weeks became the standard regimen in late 1990s. Weekly cisplatin (40 mg/m^2^) was first used in 2002, and it has become our preferred regimen in most head and neck cancer patients. Concurrent cetuximab was used in 11 patients (5%).

Figure1B shows RT techniques used over 30 years. EBRT + IB was the predominant RT technique between 1985 and 1995. After 1995, use of IB boost drastically declined from 73% (1991–1995) to 30% (1996–2000). The last brachytherapy implant was performed in 2001. As 3D conformal RT was being implemented in the 1990s, definitive EBRT increased from 27% (1991–1995) to 70% (1996–2000) to 83% (2001–2005). IMRT + SIB for BOT SCC was first introduced in our department in April of 2003 and over time it has become the predominant RT technique. The use of IMRT for BOT SCC increased from 14% (2003–2005) to 94% (2006–2011).

### Evolution of imaging techniques

Prior to 1990, before routine use of computed tomography (CT) scanners, EBRT was delivered using 2D treatment planning. After 1990, 3D conformal RT was implemented along with introduction of CT for diagnosis, simulation, and treatment planning. In parallel with the use of CT, utilization of definitive EBRT increased from 27% to 70% to 83% from 1991 to 2005. Subsequently, all radiotherapy (RT) cases utilized CT for treatment planning. Pretreatment positron emission tomography (PET)/CT was first utilized in 2003, and its use for diagnosis and treatment planning subsequently increased from 0% (before 2003) to 11% (2001–2005) to 82% (2006–2011).

### Treatment outcomes

The median follow-up of living patients was 6 years (range, 0.3–28 years). The overall Kaplan–Meier estimated that 2- and 5-year LC, RC, and LRC rates were 86%, 89%, 79% and 80%, 84%, 73%, respectively (Table4). Overall, there were 69 treatment failures and 139 deaths. The 2- and 5-year DMFS, DFS, and OS rates were 92%, 73%, 69%, and 89%, 67%, 51%, respectively.

**Table 4:** Overall treatment outcomes

LC	
No. of failures	37
2 years	86%
5 years	80%
RC	
No. of failures	28
2 years	89%
5 years	84%
LRC	
No. of failures	52
2 years	79%
5 years	73%
DMFS	
No. of DM	23
2 years	92%
5 years	89%
DFS	
No. of failures	69
2 years	73%
5 years	67%
OS	
No. of deaths	139
2 years	69%
5 years	51%

LC, local control; RC, regional control; LRC, locoregional control; DMFS, distant metastases-free survival; OS, overall survival; DFS, disease-free survival.

Clinical outcomes of the three predominant treatment modalities for stage III/IV patients were compared (Table5). The 5-year LRC and OS rates for definitive chemoRT versus RT alone were 78% versus 61% and 62% versus 36%. The 5-year LRC and OS rates for surgery followed by postop RT versus RT alone were 84% versus 61% and 62% versus 36%. There was no significant difference in treatment outcomes between surgery followed by postoperative RT and definitive chemoRT.

**Table 5:** Outcomes stratified by treatment modality for stage III–IV patients

	Definitive chemoRT (*n* = 96)	Definitive RT (*n* = 69)	Surgery + RT (*n* = 45)	Log-rank comparisons (HR, 95% CI, *P*-value)
LC				
No. of LF	17	11	6	RT vs. CRT (NS)
2 years	83%	83%	92%	S + RT vs. CRT (NS)
5 years	81%	80%	86%	RT vs. S + RT (NS)
LRC				
No. of LRF	20	22	7	RT vs. CRT (HR 1.6, CI 1.1–3.1, *P* = 0.04)
2 years	80%	66%	90%	S + RT vs. CRT (NS)
5 years	78%	61%	84%	RT vs. S + RT (HR 2.6, CI 1.2–5.4, *P* = 0.02)
DMFS				
No. of DM	11	7	5	RT vs. CRT (NS)
2 years	91%	87%	97%	S + RT vs. CRT (NS)
5 years	88%	87%	91%	RT vs. S + RT (NS)
DFS				
No. of failures	29	28	9	RT vs. CRT (NS)
2 years	73%	57%	88%	S + RT vs. CRT (NS)
5 years	69%	52%	82%	RT vs. S + RT (HR 2.6, CI 1.3–4.9, *P* = 0.01)
OS				
No. of deaths	43	61	21	RT vs. CRT (HR 1.7, CI 1.2–2.7, *P* < 0.01)
2 years	74%	51%	80%	S + RT vs. CRT (NS)
5 years	62%	36%	62%	RT vs. S + RT (HR 2.1, CI 1.4–3.3, *P* < 0.01)

HR, hazard ratio for failure or death; CI, 95% confidence interval; CRT, definitive chemoRT; S + RT, surgery + RT; RT, definitive RT; NS, not statistically significant; LC, local control; RC, regional control; LRC, locoregional control; DMFS, distant metastases-free survival; OS, overall survival; DFS, disease-free survival.

On comparison of the three predominant RT techniques for stage III/IV patients, IMRT with SIB was associated with significantly improved LRC (HR 3.2), DFS (HR 3.4), and OS (HR 3.0) when compared to conventional EBRT (Table6). The 5-year LRC, DFS, and OS rates for IMRT + SIB versus conventional EBRT were 84% versus 61%, 78% versus 45%, and 72% versus 31%. In addition, IMRT + SIB had significantly improved OS (HR 1.8) compared to EBRT + IB. The 5-year OS rate for IMRT with SIB versus EBRT plus IB was 72% versus 49%. There was no significant difference in treatment outcomes between conventional EBRT and EBRT + IB.

**Table 6:** Outcomes stratified by RT technique for stage III–IV patients

	EBRT + IB (*n* = 57)	EBRT (2D/3D) (*n* = 49)	IMRT + SIB (*n* = 45)	Log-rank comparisons (HR, 95% CI, *P*-value)
LC				
No. of LF	12	8	5	EBRT vs. EBRT + IB (NS)
2 years	81%	80%	90%	EBRT vs. IMRT + SIB (NS)
5 years	79%	80%	86%	EBRT + IB vs. IMRT + SIB (NS)
LRC				
No. of LRF	17	16	6	EBRT vs. EBRT + IB (NS)
2 years	74%	61%	87%	EBRT vs. IMRT + SIB (HR 3.2, CI 1.4–7.4, *P* < 0.01)
5 years	69%	61%	84%	EBRT + IB vs. IMRT + SIB (NS)
DMFS				
No. of DM	6	7	3	EBRT vs. EBRT + IB (NS)
2 years	90%	84%	95%	EBRT vs. IMRT + SIB (NS)
5 years	90%	79%	92%	EBRT + IB vs. IMRT + SIB (NS)
DFS				
No. of failures	21	23	8	EBRT vs. EBRT + IB (NS)
2 years	67%	48%	85%	EBRT vs. IMRT + SIB (HR 3.4, CI 1.7–6.9, *P* < 0.01)
5 years	62%	45%	78%	EBRT + IB vs. IMRT + SIB (NS)
OS				
No. of deaths	49	40	11	EBRT vs. EBRT + IB (NS)
2 years	61%	45%	82%	EBRT vs. IMRT + SIB (HR 3.0, CI 1.8–5.3, *P* < 0.001)
5 years	49%	31%	72%	EBRT + IB vs. IMRT + SIB (HR 1.8, CI 1.1–3.2, *P* = 0.04)

HR, hazard ratio for failure or death; CI, 95% confidence interval; EBRT, conventional external beam radiotherapy; EBRT + IB, conventional external beam radiotherapy plus interstitial brachytherapy; IMRT + SIB, intensity-modulated radiotherapy with simultaneous integrated boost; NS, not statistically significant. LC, local control; RC, regional control; LRC, locoregional control; DMFS, distant metastases-free survival; OS, overall survival; DFS, disease-free survival.

To control for the effects of systemic chemotherapy, subset analysis was performed comparing outcomes of the three predominant RT techniques for stage III/IV patients in the setting of concurrent chemotherapy. As demonstrated in Table7, chemoIMRT with SIB was associated with significantly improved LRC (HR 3.0), DFS (HR 3.1), and OS (HR 2.2) compared to conventional chemoEBRT. The 5-year LRC, DFS, and OS rates for chemoIMRT + SIB versus conventional chemoEBRT were 84% versus 62%, 78% versus 45%, and 72% versus 39%.

**Table 7:** Outcomes stratified by RT technique for stage III–IV patients treated with concurrent chemotherapy

	EBRT + IB (*n* = 15)	EBRT (2D/3D) (*n* = 23)	IMRT + SIB (*n* = 44)	Log-rank comparisons (HR, 95% CI, *P*-value)
LC				
No. of LF	3	6	5	EBRT vs. EBRT + IB (NS)
2 years	87%	69%	89%	EBRT + IB vs. IMRT + SIB (NS)
5 years	87%	69%	86%	EBRT vs. IMRT + SIB (NS)
LRC				
No. of LRF	3	8	6	EBRT vs. EBRT + IB (NS)
2 years	87%	62%	87%	EBRT + IB vs. IMRT + SIB (NS)
5 years	87%	62%	84%	EBRT vs. IMRT + SIB (HR 3.0, CI 1.0–9.6, *P* = 0.03)
DMFS				
No. of DM	2	4	3	EBRT vs. EBRT + IB (NS)
2 years	93%	83%	95%	EBRT + IB vs. IMRT + SIB (NS)
5 years	93%	73%	92%	EBRT vs. IMRT + SIB (NS)
DFS				
No. of failures	4	12	8	EBRT vs. EBRT + IB (NS)
2 years	80%	50%	85%	EBRT + IB vs. IMRT + SIB (NS)
5 years	80%	45%	78%	EBRT vs. IMRT + SIB (HR 3.1, CI 1.2–7.8, *P* < 0.01)
OS				
No. of deaths	11	17	11	EBRT vs. EBRT + IB (NS)
2 years	73%	57%	82%	EBRT + IB vs. IMRT + SIB (NS)
5 years	67%	39%	72%	EBRT vs. IMRT + SIB (HR 2.2, CI 1.1–4.7, *P* = 0.02)

HR, hazard ratio for failure or death; CI, 95% confidence interval; EBRT, conventional external beam radiotherapy; EBRT + IB, conventional external beam radiotherapy plus interstitial brachytherapy; IMRT + SIB, intensity-modulated radiotherapy with simultaneous integrated boost; NS, not statistically significant. LC, local control; RC, regional control; LRC, locoregional control; DMFS, distant metastases-free survival; OS, overall survival; DFS, disease-free survival.

### Complication

Table8 shows the long-term complication rates associated with RT for stage III/IV patients. Severe ≥grade 3 adverse events occurred in 25 patients (17%). Of these patients, 12 were treated with EBRT + IB, seven with conventional EBRT, and six with IMRT + SIB. There were no significant differences in rates of overall or specific ≥grade 3 toxicities among treatment groups. Severe dysphagia or pharyngeal dysfunction occurred in 11 patients (7%), resulting in prolonged use of gastrostomy tube and/or chronic aspiration pneumonia. Osteonecrosis of the mandible occurred in seven patients (5%) and soft tissue necrosis occurred in five patients (3%). Other ≥grade 3 adverse events included severe chronic pain, xerostomia, and trismus, etc.

**Table 8:** Incidence and rate of ≥grade 3 late toxicity

≥Grade 3 late toxicity, *n* (%)	EBRT + IB (*n* = 57)	EBRT (2D/3D) (*n* = 49)	IMRT + SIB (*n* = 45)	*P*-value
Dysphagia or pharyngeal dysfunction (severely altered eating/swallowing; chronic aspiration; long-term gastrostomy tube dependence)	4 (7%)	3 (6%)	4 (9%)	NS
Osteonecrosis of mandible (severe symptoms)	5 (9%)	1 (2%)	1 (2%)	NS
Other toxicity^1^	3 (5%)	3 (6%)	1 (2%)	NS
Overall toxicity	12 (21%)	7 (14%)	6 (13%)	NS

EBRT + IB, conventional external beam radiotherapy plus interstitial brachytherapy.

1 Severe chronic pain, head and neck soft tissue necrosis/fistula, severe xerostomia.

## Discussion

Curative treatment for BOT SCC at our institution has undergone several major paradigm shifts in the last three decades. During the 1980s to 1990s, a nonsurgical approach of EBRT + IB with planned neck dissection was the primary treatment modality for locoregional advanced disease. Subsequently, in the early 2000s, with randomized control trials demonstrating significant improvements in LC and survival with concurrent chemotherapy 13,14, our institution established chemoRT as the standard first-line therapy for patients with advanced BOT SCC 18. In accordance with published results, our cohort of definitive chemoRT patients had significantly improved LRC and OS compared to definitive RT patients. The addition of chemotherapy resulted in a benefit of 17% in LRC that translated into survival advantage.

Surgery with postoperative RT had 5-year LRC, DFS, and OS rates of 84%, 82%, and 62%. Karatzanis et al. 19. also reported 5-year LC, disease-specific survival (DSS), and OS rates of 86%, 63%, and 47% with primary surgery plus postoperative RT. There was no significant difference in treatment outcomes of surgery plus postoperative RT versus definitive chemoRT, although the postoperative RT group appeared numerically better in terms of LC, LRC, and DFS. However, there were significantly fewer T3/T4 tumors in the surgery plus postoperative RT cohort compared to the primary RT cohort. In our experience, combined modality approaches generally provide high local RC rates but are associated with suboptimal functional outcomes. Despite excellent disease control, surgery plus postoperative RT was not the treatment modality used for most advanced cancers at our institution due to concerns with impaired speech and swallowing outcomes. We have favored concurrent chemoRT, which provides near equivalent cure rates as surgery, but with less severe toxicity, improved organ function preservation, and improved quality of life 1,11,12,20–22.

In terms of RT technique, our preferred choice for decades had been combination EBRT with interstitial brachytherapy boost 9. In our experience using EBRT + IB for treatment of stage III/IV patients, the 5-year LC, DFS, and OS rates were 79%, 62%, and 49%, respectively. This is comparable to clinical experience at other centers. The Massachusetts General Hospital reported a 5-year LC, DFS, and OS rates of 78%, 54%, and 62% for all stage groups 23. Harrison et al. reported a 5-year LC, DFS, and OS rates of 89%, 80%, and 86% for all stages 24. Gibbs et al. reported 5-year LC and OS rates of 82% and 66% 10.

In subsequent years, RT techniques have increasingly shifted to EBRT with external beam boost. This coincided with the adoption of altered fractionation schedules, such as hyperfractionation and accelerated fractionation, in definitive EBRT 7,22. More recently, IMRT-simultaneous integrated boost (IMRT + SIB) with concurrent chemotherapy has been adopted as a radiobiologically sound and clinically effective means of delivering accelerated RT 17,25–27. The principle advantage of IMRT + SIB is that it integrates accelerated fractionation and dose escalation with concurrent chemotherapy, thereby delivering a higher daily dose to gross tumor. In addition, randomized data and institutional reports have confirmed the benefit of IMRT in reducing acute and late toxicity such as xerostomia in oropharygneal cancers 25,28. Potential disadvantages of IMRT include increased risk for a marginal miss due to sharp dose drop-off, increased dose inhomogeneity, increased integral dose, and increased costs.

Our clinical outcomes of IMRT + SIB with concurrent chemotherapy for stage III/IV patients show an excellent 5-year LRC, DFS, and OS rates of 84%, 78%, and 72%. Our findings are supported by published chemotherapy plus IMRT experience at other centers. In general, published reports show favorable results with IMRT. Lawson et al. reported a 2-year LC and OS rates of 90% and 92% in 34 patients with SCC of BOT 18. Mendenhall et al. reported institutional results of definitive IMRT for oropharyngeal cancer, with the majority of primary sites being BOT. In this series, 90% of patients had stage III–IV disease and 36% had T3–T4 primary tumors. The investigators reported a 5-year LRC rate of 72% for stage III, 94% for stage IVA, 71% for stage IVB disease, an overall 5-year cause-specific survival rate of 85% and 5-year OS rate of 76% 22. Lee et al. examined a cohort of 55 patients with oropharyngeal cancer, of which 51% were BOT SCC (15). They demonstrated that IMRT with chemotherapy resulted in lower toxicity without compromising clinical efficacy compared to conventional chemoRT plus concomitant boost, with a reported 3-year locoregional progression-free survival rate of 92%, DFS of 82%, and OS rate of 91%.

In our series, locoregional failure remains the predominant pattern of failure. At our institution, accelerated IMRT + SIB with concurrent chemotherapy has demonstrated excellent LRC for advanced SCC of BOT. This treatment regimen is associated with significantly improved LRC, DFS, and OS compared to conventional EBRT or EBRT + IB. In a separate analysis to control for differences in stage, chemotherapy usage, and the influence of planned neck dissection, the comparison between radiation techniques were restricted to stage III or IV patients who received concurrent chemotherapy and RC assessment prior to neck dissection (data not shown). In this analysis, IMRT-SIB was also associated with superior LRC, DFS, and OS compared to conventional RT techniques in the context of concurrent chemotherapy.

There are several potential reasons for the improved outcomes seen with IMRT + SIB. Published clinical trials and retrospective institutional reports have demonstrated that improvements in LRC can be attributed to concurrent chemotherapy and to accelerated fractionation 11–14. In all, 44 of 45 patients in our IMRT + SIB cohort received concurrent chemotherapy. On the other hand, chemotherapy was generally not given with brachytherapy for fear of excessive toxicity, whereas chemotherapy can be given with IMRT without causing excessive toxicity. Only 15 of 57 patients in the EBRT plus IB cohort and 23 of 49 patients in the conventional EBRT cohort received concurrent chemotherapy. In addition, patients undergoing brachytherapy had a 2- to 4-week RT treatment break between completion of EBRT and implant, potentially allowing for accelerated repopulation of tumor. Regarding fractionation schedules, all patients in the EBRT plus IB group received standard fractionation for EBRT and 45 of 49 patients in the EBRT alone group received standard fractionation. Imaging techniques may have contributed to improved clinical outcomes. The use of PET/CT for diagnosis and treatment planning has revolutionized the management of head and neck cancer 29. At our institution, PET/CT is used for staging and RT target delineation. PET/CT-based planning has become our standard practice for definitive RT cases. This is supported by studies demonstrating benefits of PET in contouring primary tumor and lymph nodes 30,31. In these studies, the PET-defined GTV was smaller and more accurate than the CT-defined GTV, and closer to the tumor volume at pathologic analysis.

Our complication rates are generally consistent with the published complication rates associated with each RT technique; however, the rate of osteonecrosis in our EBRT + IB series was slightly higher than the published complication rates of brachytherapy 10,24. On the other hand, RTOG 00-22 reported a 6% rate of osteonecrosis using IMRT 25, compared to our rate of 2% using IMRT. There was no significant difference in severe complication rates among different RT techniques. While the main reported benefit of IMRT is reduction of xerostomia 25,28, this benefit was difficult to confirm in our series as we only observed three cases of grade 3 late xerostomia. The most common nonxerostomia toxicity in our series was pharyngeal toxicity, with maximal toxicity observed at 6, 12, 18, and 24 months from start of RT. The majority of grade ≥2 toxicities were observed within the first year from start of RT, and late toxicity rates declined over time. Six patients had new or continuing ≥grade 3 toxicity at 15 or more months from the start of RT, including three cases of osteoradionecrosis 25.

The major limitation of our study is the lack of human papillomavirus (HPV) status in this cohort. The clinical outcomes seen with chemotherapy and IMRT + SIB may be strongly influenced by an increasing proportion of HPV-positive tumors, which demonstrates improved treatment response, DFS, and OS 32. In our cohort, HPV testing was started in 2005, and results are available for 24 patients. With the availability of HPV testing, de-escalated RT or less intense chemotherapy may provide a suitable alternative strategy for patients with HPV-positive oropharyngeal cancer.

In conclusion, this study demonstrates that the evolution of treatment modality and RT techniques for BOT SCC has been driven by incorporation of functional preservation, accelerated fractionation, technical advancements, and concurrent chemotherapy. This evolution is associated with excellent LRC and survival outcomes.

## Conflict of Interest

None declared.

## Supporting Information

**Table S1.** Outcomes stratified by RT technique for stage III?IV patients treated with concurrent chemotherapy. **Table S2.** Incidence and rate of ?grade 3 late toxicity. **Table S3.** Kaplan?Meier estimate of outcomes by age groups (decades).
