# Supplementary material for: Clinical outcomes associated with evolving treatment modalities and radiation techniques for base-of-tongue carcinoma: thirty years of institutional experience
Source: Cancer Med. 2015 Jan 26;4(5):651–60. doi: 10.1002/cam4.364 (PMC4430258; doi:10.1002/cam4.364)
Supplement: Supplementary file 1 — Table S1. Outcomes stratified by RT technique for stage III?IV patients treated with concurrent chemotherapy. Table S2. Incidence and rate of ?grade 3 late toxicity. Table S3. Kaplan?Meier estimate of outcomes by age groups (decades). [file cam40004-0651-sd1.doc]

Supplemental Table 1. Outcomes stratified by RT technique for stage III-IV patients treated with concurrent chemotherapy

|  |  |  |  |  |  |
| --- | --- | --- | --- | --- | --- |
|  |  | **EBRT + IB** | **EBRT (2D/3D)** | **IMRT + SIB** |  |
|  |  | **(n=15)** | **(n=23)** | **(n=44)** | **Log-rank comparisons (HR, 95% CI, p-value)** |
| **LC** | # of LF | 3 | 6 | 5 | EBRT vs EBRT+IB (NS) |
|  | 2 yr | 87% | 69% | 89% | EBRT+IB vs IMRT+SIB (NS) |
|  | 5 yr | 87% | 69% | 86% | EBRT vs IMRT+SIB (NS) |
| **LRC** | # LRF | 3 | 8 | 6 | EBRT vs EBRT+IB (NS) |
|  | 2 yr | 87% | 62% | 87% | EBRT+IB vs IMRT+SIB (NS) |
|  | 5 yr | 87% | 62% | 84% | EBRT vs IMRT+SIB (HR 3.0, CI 1.0 to 9.6, p=0.03) |
| **DMFS** | # of DM | 2 | 4 | 3 | EBRT vs EBRT+IB (NS) |
|  | 2 yr | 93% | 83% | 95% | EBRT+IB vs IMRT+SIB (NS) |
|  | 5 yr | 93% | 73% | 92% | EBRT vs IMRT+SIB (NS) |
| **DFS** | # of failures | 4 | 12 | 8 | EBRT vs EBRT+IB (NS) |
|  | 2 yr | 80% | 50% | 85% | EBRT+IB vs IMRT+SIB (NS) |
|  | 5 yr | 80% | 45% | 78% | EBRT vs IMRT+SIB (HR 3.1, CI 1.2 to 7.8, p<0.01) |
| **OS** | # of deaths | 11 | 17 | 11 | EBRT vs EBRT+IB (NS) |
|  | 2 yr | 73% | 57% | 82% | EBRT+IB vs IMRT+SIB (NS) |
|  | 5 yr | 67% | 39% | 72% | EBRT vs IMRT+SIB (HR 2.2, CI 1.1 to 4.7, p=0.02) |

HR= hazard ratio for failure or death; CI= 95% confidence interval; EBRT= conventional external beam radiotherapy; EBRT+IB= conventional external beam radiotherapy plus interstitial brachytherapy; IMRT+SIB= intensity modulated radiotherapy with simultaneous integrated boost; NS=not statistically significant

Supplemental Table 2. Incidence and rate of ≥ Grade 3 late toxicity

|  |  |  |  |  |  |
| --- | --- | --- | --- | --- | --- |
|  | **EBRT**  **+ IB (n=57)** | **EBRT (2D/3D) (n=49)** | **IMRT**  **+ SIB (n=45)** | **p-value** |  |
| **≥ Grade 3 late toxicity** | n (%) | n (%) | n (%) |  |  |
| Dysphagia or pharyngeal dysfunction (severely altered eating/swallowing; chronic aspiration; long-term gastrostomy tube dependence) | 4 (7%) | 3 (6%) | 4 (9%) | NS |  |
| Osteonecrosis of mandible (severe symptoms) | 6 (11%) | 1 (2%) | 1 (2%) | NS |  |
| Other toxicity* | 3 (5%) | 3 (6%) | 1 (2%) | NS |  |
| Overall toxicity | 13 (23%) | 7 (14%) | 6 (13%) | NS |  |
| * severe chronic pain, head and neck soft tissue necrosis/fistula, severe xerostomia | | | | |  |

Supplemental table 3. Kaplan-Meier estimate of outcomes by age groups (decades)

|  |  |  |  |  |  |  |
| --- | --- | --- | --- | --- | --- | --- |
|  |  | **40s** | **50s** | **60s** | **70s** |  |
|  |  | **(n=30)** | **(n=86)** | **(n=80)** | **(n=27)** | **Log-rank comparisons (HR, 95% CI, p-value)** |
| **LC** | # of LF | 8 | 12 | 12 | 2 | NS |
|  | 2 yr | 89% | 89% | 85% | 89% |  |
|  | 5 yr | 73% | 82% | 82% | 89% |  |
| **LRC** | # LRF | 9 | 20 | 15 | 5 | NS |
|  | 2 yr | 85% | 80% | 80% | 78% |  |
|  | 5 yr | 70% | 72% | 78% | 78% |  |
| **DMFS** | # of DM | 5 | 4 | 8 | 4 | NS |
|  | 2 yr | 96% | 95% | 90% | 87% |  |
|  | 5 yr | 86% | 95% | 88% | 87% |  |
| **DFS** | # of failures | 10 | 24 | 22 | 9 | NS |
|  | 2 yr | 85% | 75% | 73% | 67% |  |
|  | 5 yr | 66% | 68% | 69% | 67% |  |
| **OS** | # of deaths | 16 | 40 | 53 | 23 | **40s vs. 70s (HR 0.4, CI 0.2 to 0.8, p<0.01)** |
|  | 2 yr | 73% | 72% | 74% | 52% | **50s vs. 70s (HR 0.5, CI 0.2 to 0.8, p<0.01)** |
|  | 5 yr | 61% | 58% | 50% | 36% |  |
